# Prevalence of Long COVID in *Mycobacterium tuberculosis-*exposed groups

**DOI:** 10.1016/j.jctube.2026.100610

**Published:** 2026-04-25

**Authors:** Ariana R. Cardenas-Jara, Asiko Ongaya, Clement Shiluli, Lourdes B. Ramos, Liz C. Senador, Juan A. Flores, Bernard N. Kanoi, Josephine F. Reijneveld, Angel Ruvalcaba, Danny Perez, Paul Waiganjo, Cecilia S. Lindestam-Arlehamn, Timothy J. Henrich, Michael J. Peluso, Segundo R. Leon, Jesse Gitaka, Sara Suliman

**Affiliations:** a Division of Experimental Medicine, University of California, San Francisco, CA, USA; b Escuela Profesional de Tecnología Médica, Universidad Privada San Juan Bautista, Lima, Peru; c Centre for Respiratory Diseases Research, Kenya Medical Research Institute, Nairobi, Kenya; d Centre for Research in Infectious Diseases, Mount Kenya University, Thika, Kenya; e Center for Vaccine Innovation, La Jolla Institute for Immunology, La Jolla, CA, USA; f Chan Zuckerberg Biohub, San Francisco, CA, USA; g The UCSF-Gladstone Institute for Genomic Immunology, San Francisco, CA, USA; h Division of HIV, Infectious Diseases, and Global Medicine, University of California, San Francisco, San Francisco, CA, USA; i Center for Vaccine Research, Department of Infectious Disease Immunology, Statens Serum Institut, Copenhagen, Denmark; j Instituto de Investigación en Salud Global, Universidad Privada San Juan Bautista, Lima, Peru; k Department of Parasitology, Institute of Tropical Medicine, Nagasaki University, Nagasaki, Japan

**Keywords:** COVID-19, post-acute sequelae of SARS-CoV-2 infection, post-COVID condition, Long COVID, Latent tuberculosis

## Abstract

**Objectives:**

Long COVID (LC), i.e. the persistence of new or worsening symptoms for 3 months after Severe Acute Respiratory Syndrome of Coronavirus 2 (SARS-CoV-2) infection, is an emerging global health burden. The prevalence of LC remains poorly characterized in low- and middle-income countries (LMICs), where other respiratory diseases, like tuberculosis (TB), are prevalent. We aimed to address this gap in *Mycobacterium tuberculosis (Mtb)*-exposed populations in Peru.

**Methods:**

We recruited people with TB (n = 36) and their asymptomatic household contacts (n = 63) in Peru. We collected clinical data using a questionnaire adapted from a United States-based study of LC. Participants were recruited within 2 years of SARS-CoV-2 diagnosis.

**Results:**

In Peru, 41% participants reported LC symptoms. The most common LC symptoms were neurological (e.g., headache) and musculoskeletal (e.g., back pain). We did not detect an association between TB disease or *Mtb* infection and LC at this sample size. However, the quality-of-life dimensions worsened during the post-COVID period.

**Conclusions:**

LC prevalence in Peru aligns with global trends, underscoring significant health burdens. Those with LC reported high levels of musculoskeletal and neurological symptoms, highlighting the need for long-term follow-up and larger studies in different geographic settings to dissect the impact of TB comorbidity on LC.

## Highlights

• Long COVID (LC) has been poorly studied in low and middle-income countries (LMICs).
• The impact of co-infections, such as *M. tuberculosis*, on LC remains unclear.
• The prevalence of LC in Peru is high, with neurological and musculoskeletal symptoms.
• TB disease or *M. tuberculosis* infection was not associated with LC status in Peru.
• Kenyan healthcare workers reported no symptoms of Long COVID.

## 1 Introduction

Long COVID (LC) is defined as the persistence or development of new or worsening symptoms that continue to be present at least 3 months after infection with severe acute respiratory syndrome coronavirus 2 (SARS-CoV-2) [1]. Intensive efforts are now underway to identify the etiology of this condition. Prior work has identified host-related risk factors for developing LC, including female sex [2] and the presence of certain comorbidities, such as obesity, diabetes, and the presence or reactivation of chronic viral infections [3].

LC has been identified in almost all regions of the world affected by COVID-19. To date, there is limited data available from low- and middle-income countries (LMICs), since over 90% of LC studies have been conducted in high and upper-middle-income countries [4]. Furthermore, key questions remain about the impact of other co-infections, which may be vastly more prevalent in LMICs, particularly *Mycobacterium tuberculosis* (*Mtb*) infection, the causative agent of tuberculosis (TB) disease, estimated to affect 10 million people annually [5]. Mouse model studies showed that pre-existing *Mtb* infection decreased SARS-CoV-2 viral loads and weight loss, suggesting a paradoxical protective role of *Mtb* against SARS-CoV-2 [6]. However, it remains unknown whether *Mtb*-driven chronic inflammation alters the trajectory of LC, either by facilitating viral clearance or, conversely, by exacerbating the persistent inflammatory dysregulation characteristic of LC. Indeed, more studies are urgently needed to define the interaction between *Mtb* exposure and LC risk in human populations. Therefore, the aim of this study was to characterize the prevalence of LC in LMICs, particularly among populations exposed to *Mtb*.

## 2 Methods

### Ethical approval

This study was approved by San Juan Bautista Private University Ethics Committee (N°705-2022-CIEI-UPSJB) in Peru, Mount Kenya Ethics (MKU/ERC/2252) in Kenya, and the University of California, San Francisco (IRB no. 22–37759). We conducted the study in accordance with the Good Clinical Practice guidelines. Written informed consent was obtained from all participants before recruitment.

### 2.2 Study design and Participants

**Lima, Peru:** We enrolled 99 adult participants, 36 persons diagnosed with pulmonary tuberculosis, and 63 household contacts (HHCs) between January 2023 and May 2024. Recruitment was conducted at 35 health centers and the Maria Auxiliadora Hospital in Southern Lima, Peru. All participants had a documented history of prior SARS-CoV-2 infection, confirmed either by a positive molecular or antigen test, or inferred from the development of symptoms within two weeks of cohabitation with a person with COVID-19. HHCs were contacted through the index cases (participants with TB) and were defined as persons living with the TB patient in the same housing unit. After the recruitment, HHCs were stratified by the QuantiFERON-TB-Gold plus (QFT) test results as described below. Participants with active TB were diagnosed either by a positive sputum smear test or clinical criteria (TB symptomatology and radiologic signs), according to the Peruvian National TB guidelines [7]. All persons with TB undergoing treatment were invited to participate unless they had a confirmed diagnosis of HIV or any form of drug-resistant TB, information we obtained from their treatment cards and clinical records. The interviews were conducted in the household unit at recruitment, two, and four months after the initial visit.

**Nairobi, Kenya:** To make a comparative analysis, we enrolled 202 participants from Mbagathi Hospital in Nairobi, Kenya. We recruited healthcare workers, including medical doctors, nurses, clinical officers, pharmacists, pharmaceutical technologists, radiologists, laboratory technologists, and technicians, among others, in a cross-sectional study design from April 2023 to July 2024. We collected data and samples at a single visit. We acknowledge that as healthcare workers, this cohort represents a distinct demographic from the Peruvian community-based cohort, with potentially different health-seeking behaviors, occupational exposures, and baseline health status (healthy worker effect).

### 2.3 Data collection

In both cohorts, we collected epidemiological, clinical, and COVID-19 symptomatology data using an instrument adapted from the University of California, San Francisco-based Long-term Impact of Infection with Novel Coronavirus (LIINC) study [8], which has been used to assess the presence and severity of post-acute COVID-19 attributed symptoms and quality of life in San Francisco and has supported phenotyping for multiple studies of Long COVID pathobiology [9]. Trained interviewers administered the questionnaires. We collected demographic data, including age, sex, education level, and residence type (permanent houses made of bricks or stones, or non-permanent houses made of cardboard, mud, iron sheets, or wood). Clinical data included body mass index (BMI) and comorbidities, including autoimmune diseases, cancer, diabetes, hypertension, history of heart attack, and other lung conditions (e.g., asthma). We also collected behavioral data, including smoking habits. Alcohol use data were collected according to the WHO-approved test: Development of the Alcohol Use Disorders Identification Test (AUDIT) [10].

LC was defined as having at least one COVID-related symptom present at least 90 days following SARS-CoV-2 infection, consistent with the WHO [1] and the National Academies of Sciences, Engineering, and Medicine (NASEM) [11]. Interviews were conducted to collect information on the date of SARS-CoV-2 symptom onset, the presence of symptoms, and their duration (in days), using an adaptation of the LIINC questionnaire [8]. If the person had more than one reported COVID-19 episode, we asked for the symptoms presented during the most recent SARS-CoV-2 infection. Finally, we recorded hospitalization (admission for more than 24 h), supplemental oxygen administration, admission to an intensive care unit (ICU), and mechanical ventilation.

### 2.4 Quality of Life

Quality of life was measured using questions adapted from the EuroQoL metrics [12]. The metrics measured five domains: mobility, self-care, usual activities, pain, and depression, where one corresponded to no problems while performing the activity, and five corresponded to extreme problems or inability to perform the activity. Furthermore, we recorded self-reported quality of life scores before and after COVID-19.

### 2.5 QuantiFERON-TB-Gold plus (QFT) analysis

We collected whole blood samples from participants in 6 mL lithium heparin vacuum tubes and transported them to the laboratory at room temperature within 4 h of collection. QFT tests were performed to calculate IFN-γ concentrations (IU/mL) in contacts following the manufacturer’s instructions. Indeterminate QFT results were repeated, and if they remained indeterminate, they were excluded from the analysis.

### 2.6 Statistical analysis

We described categorical variables as frequency rates and percentages, and continuous variables as medians and interquartile ranges (IQRs). We calculated the association between categorical variables and QFT groups using the Fisher's exact test. We applied the Kruskal-Wallis test for continuous variables, with a significance threshold level of 0.05. COVID-19 symptomatology information was self-reported as the presence or absence of the symptom. If the symptoms persisted at the time of the recruitment, we calculated the duration by subtracting the symptom onset date from the interview date. Like the LIINC study, we grouped the symptoms into eight categories according to the impacted body system: cardiopulmonary, constitutional, dermatologic, gastrointestinal, genitourinary, musculoskeletal, neurologic, and upper respiratory [8]. Participants were considered within the category if they had at least one symptom from that category.

We estimated prevalence ratios (PRs) using generalized linear models (GLMs) with a log link and robust Poisson error variance. Poisson regression was applied to evaluate the development of long COVID (LC) based on symptoms present during the acute phase and persisting ≥ 10 days after diagnosis or symptom onset. The 10-day threshold was selected according to the Peruvian Ministry of Health guidelines for clinical release from COVID-19 [13]. Crude rates were calculated in the univariate model; for the multivariate analysis, we adjusted for age, sex, and BMI a priori, based on epidemiological criteria [2], [14]. Quality of life responses were summarized as frequencies and percentages. Within each TB group, quality of life before and after COVID-19 was compared using Fisher’s exact test. Quality of life scores were analyzed as continuous variables. Statistical analyses were performed using STATA (version 17), and figures were generated with R (version 4.3.0) and GraphPad Prism (version 10.3.1).

## 3 Results

### 3.1 Study participants

In the Peruvian cohort, 41% of the participants reported having Long COVID (LC) (Table 1). The sociodemographic results show that the participants in the LC group were older (39 years, IQR: 29–49) than the non-LC group (32 years, IQR: 24–41); however, this difference was not statistically significant. The LC group presented a significantly higher percentage of females (78%) in comparison to the non-LC group (34%) (p < 0.001). Furthermore, we asked participants about their alcohol and smoking habits. According to the Alcohol Use Disorders Identification Test (AUDIT) guidelines [10], the majority (88%) had a low risk of developing alcohol addiction. However, the non-LC group had more people at medium or high risk of developing alcohol addiction (19%) compared to the LC group (2.4%) (p = 0.013). Body Mass Index (BMI) was elevated in both groups, but slightly higher in the LC group; this difference was not significant. The most prevalent comorbidities were diabetes, hypertension, and other lung conditions like asthma (Table 1). However, we did not identify differences in the presence of comorbidities between the groups.

**Table 1:** Factors associated with Long COVID in the Peruvian Cohort.

	**Peru Cohort**			
**Characteristic**	**Total**	**No Long COVID n = 58**	**Long COVID** **n = 41**	**p-value†**
Age*	34 (24–48)	32 (24–––41)	39 (29–––49)	0.13
**Sex at birth**				<0.001
Male	47 (47.0)	37 (65.7)	9 (22.0)	
Female	52 (53.0)	20 (34.0)	32 (78.0)	
**Type of residence**				0.2
Non-permanent	16 (16.0)	7 (12.0)	9 (22.0)	
Permanent	83 (84.0)	51 (88.0)	32 (78.0)	
**Alcohol addiction risk**				0.013
Low risk	87 (88.0)	47 (81.0)	40 (98.0)	
Medium-High risk	12 (12.0)	11 (19.0)	1 (2.4)	
**Smoking during last year**				0.1
Yes	17 (17.0)	13 (22.0)	4 (9.8)	
No	83 (83.0)	45 (88.0)	37 (90.2)	
Body Mass Index (BMI)*	26.0 (22.3–––29.6)	25.0 (21.4–––29.3)	26.9 (24.5–––29.8)	0.1
**Comorbidities**				
**Autoimmune diseases**				>0.9
Yes	4 (4.0)	2 (3.4)	2 (4.9)	
**Cancer**				0.5
Yes	2 (2.0)	2 (3.4)	0 (0.0)	
**Diabetes**				0.2
Yes	11 (11.1)	4 (6.9)	7 (17.1)	
**Hypertension**				>0.9
Yes	10 (10.1)	6 (10.3)	4 (9.8)	
**Heart Attack**				
Yes	1 (1.0)	1 (1.7)	0 (0.0)	
**Lung problems**				0.5
Yes	12 (12.1)	6 (10.3)	6 (14.6)	
**Comorbidities**				0.4
No	70 (70.7)	43 (74.1)	27 (65.9)	
Yes, at least 1	19 (19.2)	9 (15.5)	10 (24.4)	
Yes, 2 or more	10 (10.1)	6 (10.3)	4 (9.7)	
**TB history**				
**TB status**				0.3
QFT (−)**	31 (31.3)	16 (28.0)	15 (37.0)	
QFT (+)**	32 (32.3)	17 (29.0)	15 (37.0)	
Active TB	36 (36.3)	25 (43.0)	11 (27.0)	
**Current/ Previous TB diagnosis**			0.12	
No	56 (57.0)	29 (50.0)	27 (66.0)	
Yes	43 (43.0)	29 (50.0)	14 (34.0)	
**Current /Previous Pulmonary TB treatment**			0.4	
Still under treatment	34 (79.0)	23 (79.0)	11 (79.0)	
Did not start treatment	1 (2.3)	1 (3.4)	0 (0.0)	
Did not complete treatment	1 (2.3)	1 (3.4)	0 (0.0)	
Yes, 6 months	5 (12.0)	4 (14.0)	1 (7.1)	
Yes, 9 months	2 (4.7)	0 (0.0)	2 (14.0)	
**COVID-19 history**				
**Vaccine doses**				0. 16
0	5 (5.1)	1 (1.7)	4 (9.8)	
1+	94 (94.9)	57 (98.3)	37 (90.2)	
**COVID history**				<0.001
Confirmed	56 (57.0)	23 (40.0)	33 (80.0)	
Very Likely	43 (43.0)	35 (60.0)	8 (20.0)	

*median (IQR); † Fisher’s exact test for categorical variables and Wilcoxon rank sum test for numerical variables.

**QFT (−): TB household contact with negative Quantiferon test; QFT (+): TB household contact with positive QuantiFERON TB Gold test.

We did not see a difference in vaccination history within our comparison groups (p = 0.16). However, both the LC and non-LC groups received at least 1 COVID-19 dose (94.9%), indicating high coverage. Due to the limited availability of COVID-19 tests in Peru, we diagnosed COVID-19 either by a confirmed positive antigen or molecular (PCR) test, or by the development of symptoms within 2 weeks of exposure to an index SARS-CoV-2-positive case. More than half of the participants (57%) had a COVID-19 diagnosis confirmed by a laboratory test, which had a higher rate of LC than participants diagnosed by confirmed exposure and symptomology (Table 1).

As an exploratory analysis with this limited sample size, we did not find differences in LC rates based on TB status (p = 0.3) (Table 1). Namely, we did not observe a difference in LC rates among TB contacts who have different QuantiFERON-TB Gold status (S1 Table), or when comparing the contacts to the patients with active TB (S2 Table).

### 3.2 No association between *Mtb* exposure and prevalence of long COVID

In the HHCs (n = 63), 48% persons met the criteria for LC (>90 days); however, there was no difference in LC prevalence between QFT- and QFT + groups (p > 0.9), suggesting that LC was present irrespective of *Mtb* infection (S1 Table).

**Fig. 1:**
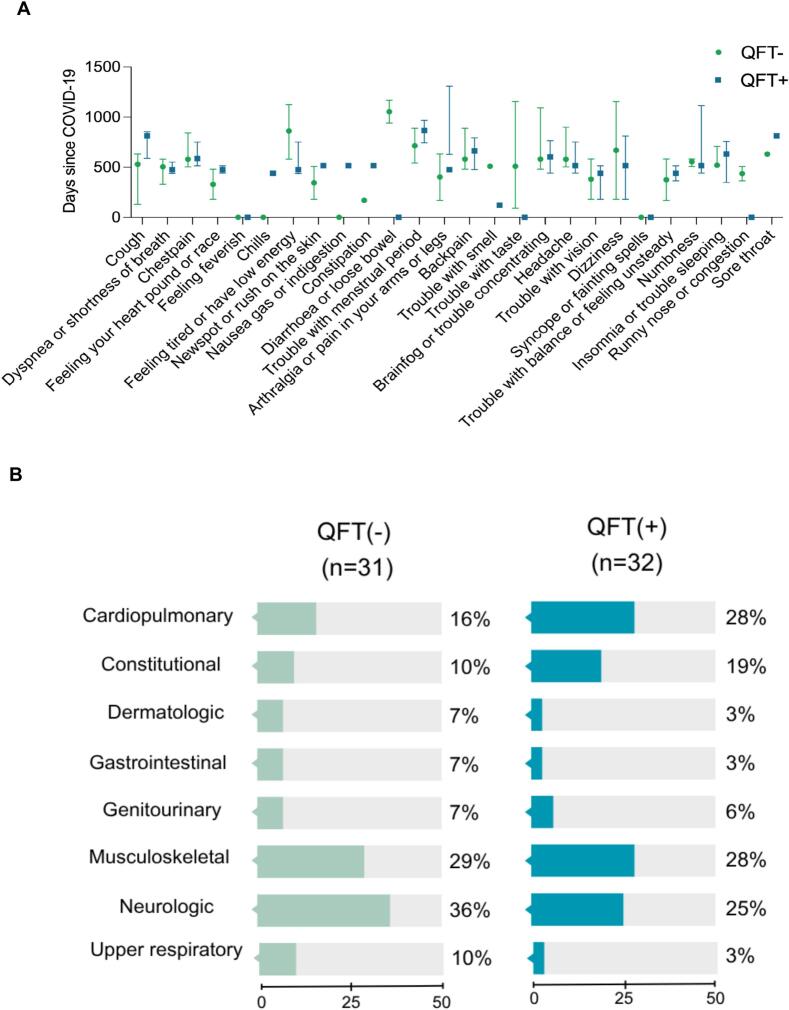
Long COVID symptoms in the Peruvian cohort. **A.** Median duration of LC symptoms by groups. **B.** Long COVID symptoms prevalence grouped by organ systems.

We analyzed LC symptoms retrospectively by duration in days. Diarrhea was the most durable symptom in the QFT- group (median [IQR]: 1054 [940–1168] days) and tiredness (861 [580–1124] days). In the QFT + group, the most durable symptoms were menstrual pain (856 [743–969] days) and cough (812 [588–854] days) (Fig. 1A). After grouping the symptoms by organ systems, we found that musculoskeletal, neurological, and cardiopulmonary symptoms were the most prevalent (Fig. 1B).

### 3.3 Influence of TB disease on long COVID

In the active TB group (n = 36), 31% of participants met the criteria for LC (S2 Table). No significant difference in LC prevalence was observed compared with HHCs (p = 0.1, S2 Table). The most frequently reported symptom categories were musculoskeletal and neurological (S1a. Fig).

### 3.4 Acute COVID-19 symptoms

Since TB status was not associated with LC (Table 1), we combined all the TB groups from the Peruvian cohort to analyze the distribution of symptoms (n = 99). The most prevalent symptoms during acute SARS-CoV-2 infection in the QFT (−) group were tiredness (77.4%), headache (64.5%), and cough (64.5%); while in the QFT (+) group were headache (81.3%), cough (78.1%), and fever (71.9%) (S3 Table).

### 3.5 Influence of COVID-19 and LC on quality of life

**Fig. 2:**
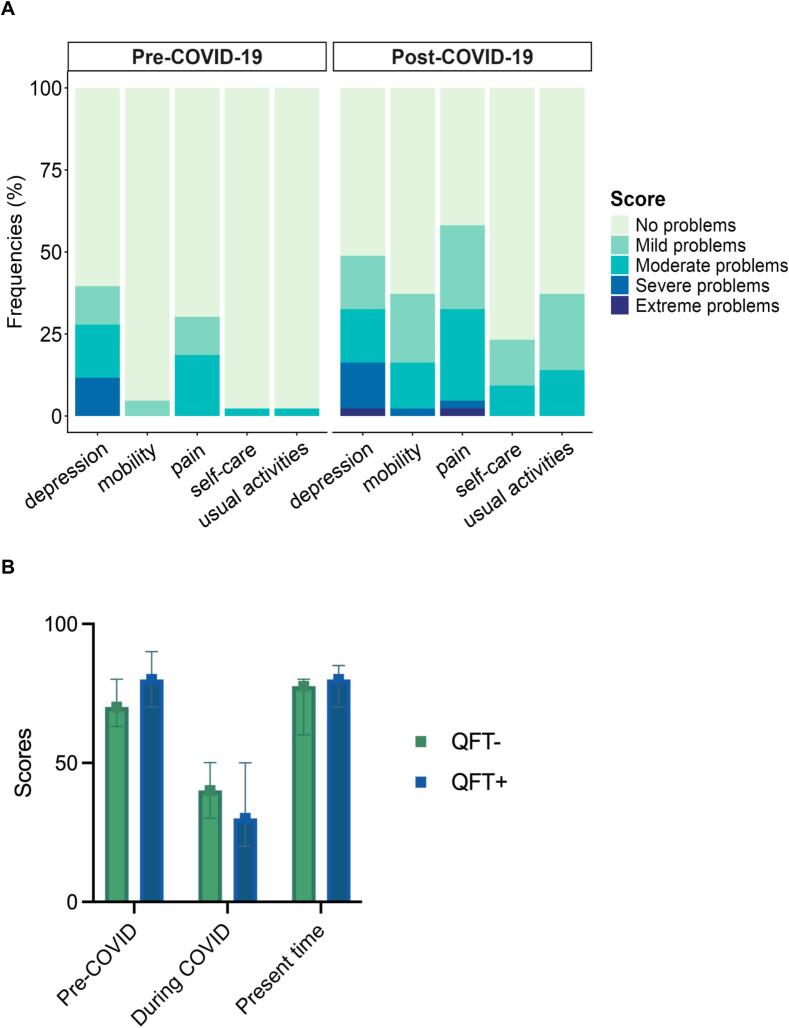
Quality of Life in the Peruvian Cohort. **A.** Pre-COVID-19, and Post-COVID-19 prevalence of discomfort while performing an activity. **B.** Self-reported health scores (0 to 100).

Next, we analyzed the impact of LC on self-reported quality of life and health scores using the EuroQoL metrics [8]. We analyzed the level of discomfort or difficulty experienced when the participants performed different activities before and after COVID-19. There was a significant increase after COVID-19 in discomfort in mobility (p = 0.02), self-care (p = 0.009), and performing usual activities like going to work or study (p < 0.001) (Fig. 2A). Interestingly, pain (p = 0.07), depression and anxiety (p = 0.9), did not show a similar trend (Fig. 2A). There was a significant difference in self-reported health scores between groups in the pre-COVID period (p = 0.037), but not during the acute (p = 0.4) or post-acute phase of COVID-19 (p = 0.2) (Fig. 2B). Similar trends were observed in the active TB group (S1b, S1c Fig.). These findings support the hypothesis that COVID-19 and LC had an impact regardless of *Mtb* infection or TB disease status.

### 3.6 Persistence and evolution of LC symptoms over time

**Fig. 3:**
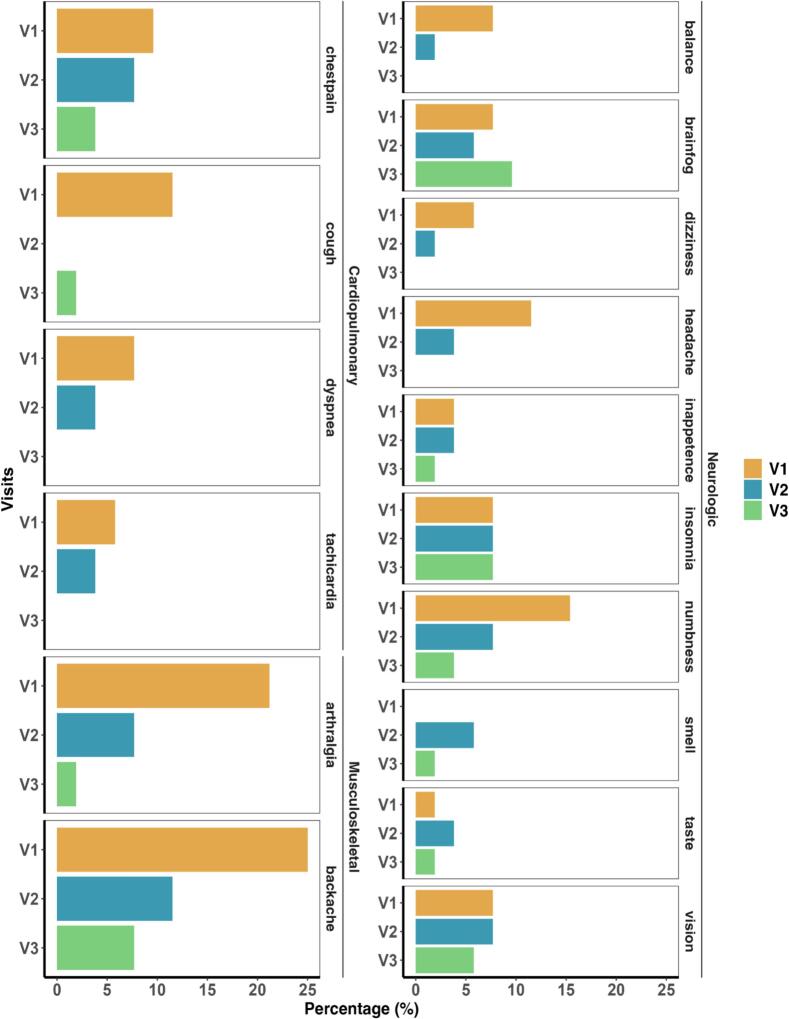
Analysis of persistence of LC symptoms at baseline (V1), after two months (V2), and after four months (V4). The most recent date of COVID-19 diagnosis ranges from 130 to 1,493 days before baseline.

We grouped all participants who completed all three follow-up visits (n = 52) to evaluate the persistence of LC symptoms over time during longitudinal follow-up visits. Symptom persistence was assessed at baseline, two months, and four months after recruitment. Since information was collected retrospectively for a prior COVID-19 episode, the time from the most recent infection to reported symptom onset varied widely, ranging from 130 to 1,493 days. Although the frequency of LC symptoms declined over time compared to the baseline, 11 of the 16 assessed symptoms (68.8%) were still reported at the final visit, particularly neurological symptoms such as brain fog and insomnia (Fig. 3).

### 3.7 The association between acute COVID-19 symptoms and LC

**Fig. 4:**
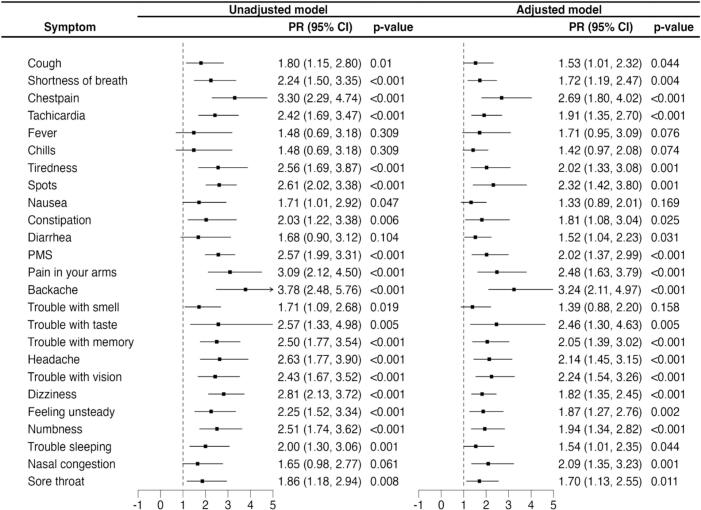
The Prevalence Ratio (PR) of LC according to the persistence of the symptoms for more than 10 days. The model on the right is adjusted by age, sex, and BMI.

Next, we evaluated whether acute COVID-19 symptoms persisting for more than 10 days were associated with LC prevalence in the entire cohort (n = 99). We calculated Prevalence Ratios (PR) and compared participants who experienced symptoms for more than 10 days to the reference group who did not experience the symptoms or experienced them for less than 10 days. Adjusting by sex, age, and body mass index (BMI), the acute symptoms most strongly associated (p < 0.001) with LC were chest pain (PR [95% confidence interval (CI)]: 2.69, [1.8–4.02]), tachycardia (1.91 [1.35–2.70]), menstrual pain (2.02 [1.37–2.99]), pain in the arms or legs (2.48[1.63–3.79]), backache (3.24 [2.11–4.97]), trouble with memory (2.05 [1.39–3.02]), headache (2.14 [1.45–3.15]), trouble with vision (2.24 [1.54–3.26]), dizziness (1.82 [1.35–2.45]), and numbness (1.94 [ CI 1.34–2.82]). We found similar trends in the unadjusted model (Fig. 4). This data suggests that specific symptom types in the acute phase of COVID-19 can predict LC incidence.

### 3.8 LC prevalence in a cross-sectional group in Kenya

We enrolled 202 healthcare workers in Kenya (S4 Table). There were 94 QFT-, and 108 QFT + participants. The median age between QFT- and QFT + groups was similar (S4 Table), and both groups had a female majority, with no difference in the sex distribution between the two groups. Similarly, alcohol use, smoking habits, and comorbidities were infrequent. All Kenyan participants received a single COVID-19 vaccine dose. All 202 participants in the Kenyan cohort were diagnosed with COVID-19 by PCR (S4 Table).

In the Kenyan cohort, the most prevalent symptoms during acute COVID-19 were tiredness (60.2%), fever (55.1%), and chills (50.5%) (S3 Table). In contrast to the Peruvian cohort, no participants in the Kenyan cohort met the criteria for LC (S4 Table). We also analyzed the durability of COVID-19 symptoms, where the median did not exceed 8 days across the different symptoms (S2 Fig.).

Using the EuroQoL metrics questions, participants experienced little or no difficulties performing activities before COVID-19. However, participants reported increased difficulties during the COVID-19 episode, particularly in mobility, self-care, and pain/discomfort (p < 0.001), which returned to baseline levels after the acute phase of infection (S3 Fig.). There was no significant difference in the self-reported health scores between QFT- and QFT + groups in the Kenyan site (S3 Fig.).

## 4 Discussion

Long COVID (LC) is a major global health concern [15]; however, the prevalence of LC in LMICs and the impact of respiratory pathogens like *Mtb* on LC risk remains unclear. Here, we apply study tools previously used to describe LC in the U.S. and report LC prevalence and risk factors in two groups in Peru and Kenya. To our knowledge, our study is among the first ones to report LC in a population highly exposed to *Mtb* in an LMIC setting.

LC prevalence was 41% in our cohort, similar to prior descriptions of LC in high-income countries. Meta-analyses studies estimated the pooled prevalence of LC to be around 36%, with lower rates in North America than in South America [16]. This data was supported by another *meta*-analysis of 48 studies, which reported that the burden of LC was 41% and varies depending on biological sex and geographical region; most of the studies included in this analysis were in North America [17].

Several hypotheses have been proposed to explain the etiology of LC. Some predictors include female sex [2], [18], older age, high body mass index [19], vaccination or prior infection [20], more severe acute disease, and comorbidities [21]. Although some data suggest persistence of SARS-CoV-2 reservoirs in tissues may be associated with LC symptoms [13], it is unclear if the detected virus is replication competent [22].

In our cohort, female sex was significantly associated with LC. A recent series of studies described an association between reduced testosterone and increased myelopoiesis and monocyte frequency in females with LC[23]. The association between medium-to-high alcohol risk and lower LC prevalence is likely confounded by sex. In our cohort, males, who had significantly lower rates of LC, were more likely to report higher alcohol consumption compared to females, who bore the burden of LC symptoms. Collectively, the high prevalence of LC in this young adult Peruvian cohort suggests the involvement of previously reported risk factors, including unclear ones, like vaccination [20].

In our cohort, TB status was not associated with LC, although this was limited by the sample sizes in the subgroup analyses. The relationship between SARS-CoV-2 and TB has been studied before; however, the impact of TB disease or exposure on COVID-19 outcomes, like LC, is underexplored. Recent studies suggest that the coexistence of *Mtb* and viruses in the lungs may increase the risk of active TB by modulating the immune system [24]. Furthermore, clinical evidence suggests that COVID-19 may predispose patients to TB disease or reactivation of latent *Mtb* infection through severe depletion and dysfunction of T-cells and uncontrolled production of pro-inflammatory cytokines [25]. Most importantly, TB disease was shown to be associated with severe COVID-19, and *Mtb/*SARS-CoV-2 co-infection may lead to exacerbated disease [25]. A significant challenge in dissecting this interaction is the substantial overlap between LC and TB symptoms, such as fatigue, dyspnea, and cough, which are hallmark features of both conditions. In our active TB cohort, distinguishing LC symptoms from the effects of tuberculosis remains clinically difficult without longitudinal pre-pandemic baselines.

Surprisingly, no LC was detected in a cohort in Kenya. This could be due to several reasons. The wide variation of LC prevalence between both cohorts could be a result of the diversity of study designs and the studied subpopulations (e.g., health-care workers). While protective measures like masking were prevalent, the complete absence of reported symptoms suggests potential unmeasured factors. These may include the 'healthy worker effect,' where active HCWs are inherently healthier than the general population, or occupational stigma, where HCWs may minimize reporting subjective symptoms like fatigue or brain fog to avoid perceived professional incapacity. Additionally, cultural differences in the interpretation of the LIINC questionnaire's symptom definitions cannot be ruled out. Furthermore, in Kenya, public health measures to protect healthcare workers led to earlier vaccination than the general population. In addition, the stress of patient care during the pandemic may have masked the prevalence of LC symptoms.

Systematic reviews reporting LC symptoms found that fatigue and dyspnea were the most frequent, with a pooled prevalence ranging from 35 to 60% [26]. The most prevalent LC symptoms in our cohort, clustered by organ system, were musculoskeletal and neurological. Another study characterizing LC symptoms by duration found that symptoms like fatigue (37%) and dyspnea (35%) were more likely to last less than 12 weeks, while fatigue (48%) and sleep disturbance (44%) were more common to last for more than 12 weeks [27]. Collectively, these studies demonstrate the persistence of musculoskeletal and neurological symptoms post-infection, highlighting the need for interventions targeting these symptom groups.

Our study recruited people with COVID-19 history from 2020 to 2023, ranging from 130 to 1,493 days after COVID-19. Although the days’ range was wide, we identified persistent neurological symptoms at the last visit, such as brain fog (9.6%), back pain, and insomnia (7.7%). A study showed that the prevalence of LC symptoms after two years of SARS-CoV-2 infection was 30%, with fatigue being the most prevalent (28%), cognitive impairments (27.6%), and pain (8.4%) [28].

COVID-19 was reported to have a lasting impact on several quality-of-life measures. A study evaluated the cognitive functioning in people with post-COVID conditions and found higher self-reported functional impairment, a lower likelihood of full-time employment, and more severe depressive symptoms that lasted for 12 months [29]. In Peru, significant differences were found in mobility, self-care, and daily activities, but no significant changes in pain, depression, and anxiety were noted before and after COVID-19. Although trends suggest an increase in depression and anxiety post-COVID [28], [30], this may be due to the social and economic burdens of the COVID-19 pandemic. These social factors confound the interpretation of causal relationships between COVID-19 and the persistence of depression and anxiety [30].

Our study has several limitations. First, our sample size is relatively small, particularly for subgroup analyses, which may limit the generalizability of our findings. Within the Peruvian cohort, the index cases and contacts may have different exposure histories to SARS-CoV-2, despite sharing the same households. Furthermore, the impact of lung pathology in TB patients on secondary exposures, such as SARS-CoV-2, is an important clinical question. However, future larger studies with detailed clinical data, including imaging and lung function tests, will address these questions in more detail. Second, LC diagnosis relies on self-reports of various symptoms, which could introduce biases such as recall or social desirability bias among healthcare workers. Third, the decision to participate could be influenced by the presence or absence of LC symptoms in ways that are not predictable. Fourth, in order to diagnose SARS-CoV-2 infection, we had to either rely on confirmed testing, which was severely limited in Lima, or a combination of confirmed exposure and symptom development, to enrich our sample size. We acknowledge the bias in this diagnostic definition. However, this reflects real-life COVID-19 diagnosis during that period. The prevalence of LC in the molecularly-confirmed group was higher in our analysis, raising the possibility that LC prevalence may be even higher in this target population. Finally, our analysis was limited to clinical symptomatology; further work could explore whether biological drivers in our cohort are similar to those in other cohorts using the same instrument, such as LIINC in the U.S. [8].

## 5 Conclusion

We identified prevalence rates of LC in Peru to be similar to global trends. We observed no statistically significant difference in LC rates based on TB infection or disease status in this cohort, though larger studies are required to rule out modest associations. Conversely, healthcare workers in Kenya did not report any LC symptoms. Our findings underscore the need for follow-up strategies for persons with COVID-19 and larger studies to explore the impact of TB on the persistence of COVID-19 symptoms.

## CRediT authorship contribution statement

**Ariana R. Cardenas-Jara:** Writing – review & editing, Writing – original draft, Visualization, Validation, Project administration, Methodology, Investigation, Formal analysis, Data curation, Conceptualization. **Asiko Ongaya:** Writing – review & editing, Writing – original draft, Visualization, Methodology, Investigation, Formal analysis, Data curation, Conceptualization. **Clement Shiluli:** Writing – original draft, Formal analysis. **Lourdes B. Ramos:** Writing – review & editing, Methodology, Formal analysis, Data curation. **Liz C. Senador:** Project administration, Methodology, Investigation, Data collection, Manuscript review. **Juan A. Flores:** Project administration, Methodology, Investigation, Data collection, Manuscript review. **Bernard N. Kanoi:** Writing – review & editing, Funding acquisition. **Josephine F. Reijneveld:** Funding acquisition, Conceptualization. **Angel Ruvalcaba:** Project administration, Conceptualization. **Danny Perez:** Methodology, Data curation. **Paul Waiganjo:** Project administration, Methodology, Investigation, Data curation. **Cecilia S. Lindestam-Arlehamn:** Writing – review & editing, Methodology, Funding acquisition, Conceptualization. **Timothy J. Henrich:** Writing – review & editing, Resources, Methodology. **Michael J. Peluso:** Writing – review & editing, Writing – original draft, Resources, Methodology. **Segundo R. Leon:** Writing – review & editing, Supervision, Project administration, Funding acquisition, Conceptualization. **Jesse Gitaka:** Writing – review & editing, Writing – original draft, Supervision, Investigation, Funding acquisition, Conceptualization. **Sara Suliman:** Writing – review & editing, Writing – original draft, Supervision, Project administration, Methodology, Investigation, Funding acquisition, Conceptualization.

## Declaration of competing interest

The authors declare that they have no known competing financial interests or personal relationships that could have appeared to influence the work reported in this paper.
