# Supplementary material for: Prevalence of Long COVID in Mycobacterium tuberculosis-exposed groups
Source: J Clin Tuberc Other Mycobact Dis. 2026 Apr 25;44:100610. doi: 10.1016/j.jctube.2026.100610 (PMC13156720; doi:10.1016/j.jctube.2026.100610)
Supplement: Supplementary Data 1 [file mmc1.docx]

**Supplementary data.**

**Table S1**. Clinical and sociodemographic characteristics in the household contacts (HHCs) in the Peru cohort.

|  | **Peru Cohort** | | |  |
| --- | --- | --- | --- | --- |
| **Characteristic** | **Total**  **n=63** | **QFT (-)****  **n=31** | **QFT (+)****  **n =32** | **p-value†** |
|  |  |  |  |  |
| **Age*** | 38 (27-50) | 40 (22-52) | 37 (29 - 46) | >0.9 |
| **Sex at birth** |  |  |  | 0.7 |
| Male | 21 (33.0) | 11 (35.5) | 10 (31.2) |  |
| Female | 42 (67.0) | 20 (64.5) | 22 (68.8) |  |
| **Type of residence** |  |  |  | 0.3 |
| Non-permanent | 11 (17.0) | 7 (22.6) | 4 (12.5) |  |
| Permanent | 52 (83.0) | 24 (77.4) | 28 (87.5) |  |
| **Alcohol addiction risk** |  |  |  | > 0.9 |
| Low risk | 56 (89.0) | 28 (90.3) | 28 (87.5) |  |
| Medium-High risk | 7 (11.0) | 3 (9.7) | 4 (12.5) |  |
| **Smoking during the last year** |  |  |  | 0.8 |
| Yes | 11 (17.0) | 5 (16.1) | 6 (18.8) |  |
| No | 89 (83.0) | 26 (83.9) | 26 (81.2) |  |
| **Body Mass Index (BMI)*** | 27.4 (24.3 - 31.2) | 28.3 (25.3 - 33.0) | 26.4 (23.7 - 30.6) | 0.2 |
| **Comorbidities** |  |  |  |  |
| **Autoimmune diseases** |  |  |  | >0.9 |
| Yes | 4 (6.3) | 2 (6.5) | 2 (6.3) |  |
| **Cancer** |  |  |  | 0.5 |
| Yes | 2 (3.2) | 0 (0.0) | 2 (6.3) |  |
| **Diabetes** |  |  |  | 0.2 |
| Yes | 5 (7.9) | 4 (12.9) | 1 (3.1) |  |
| **Hypertension** |  |  |  | >0.9 |
| Yes | 7 (11.1) | 3 (9.7) | 4 (12.5) |  |
| **Heart Attack** |  |  |  |  |
| Yes | 0 (0.0) | 0 (0.0) | 0 (0.0) |  |
| **Lung problems** |  |  |  | 0.15 |
| Yes | 8 (12.7) | 6 (19.4) | 2 (6.3) |  |
| **Comorbidities** |  |  |  | 0.066 |
| No | 45 (71.4) | 19 (61.3) | 26 (81.3) |  |
| Yes, at least 1 | 11 (17.5) | 9 (29.0) | 2 (6.3) |  |
| Yes, 2 or more | 7 (11.1) | 3 (9.7) | 4 (12.5) |  |
| **TB history** |  |  |  |  |
| **Current/ Previous TB diagnosis** | |  |  | 0.1 |
| No | 56 (89.0) | 30 (97.0) | 26 (81.0) |  |
| Yes | 7 (11.0) | 1 (3.2) | 6 (19.0) |  |
| **Current /Previous Pulmonary TB treatment** | |  |  | 0.4 |
| Still under treatment | 0 (0.0) | 0 (0.0) | 0 (0.0) |  |
| Did not start treatment | 0 (0.0) | 0 (0.0) | 0 (0.0) |  |
| Did not complete treatment | 1 (14.0) | 0 (0.0) | 1 (17.0) |  |
| Yes, 6 months | 4 (57.0) | 0 (0.0) | 4 (67.0) |  |
| Yes, 9 months | 2 (29.0) | 1(100.0) | 1 (17.0) |  |
| **COVID-19 history** |  |  |  |  |
| **Vaccine doses** | 3 (3 - 4) | 3 (3 - 4) | 4 (3 - 4) | 0.3 |
| **COVID history** |  |  |  | 0.9 |
| Confirmed | 42 (67.0) | 21 (67.7) | 21 (65.6) |  |
| Very Likely | 21 (33.0) | 10 (32.3) | 11 (34.4) |  |
| **Long COVID** |  |  |  | >0.9 |
| No | 33 (52.0) | 16 (51.6) | 17 (53.1) |  |
| Yes | 30 (48.0) | 15 (48.4) | 15 (46.9) |  |
| *median (IQR); † Fisher’s exact test for categorical variables and Kruskal-Wallis test for numerical variables.  **QFT (-): TB household contact with negative Quantiferon test; QFT (+): TB household contact with positive QuantiFERON TB Gold test. | | | | |

**Table S2**. Clinical and sociodemographic characteristics in the active TB group compared to the household contacts (HHCs) in the Peru cohort.

|  | **Peru Cohort** | | |  |
| --- | --- | --- | --- | --- |
| **Characteristic** | **Overall**  **n=99** | **HHC****  **n=63** | **ATB****  **n =36** | **p-value†** |
|  |  |  |  |  |
| **Age*** | 34 (24-48) | 38 (27-50) | 30 (24 - 40) | 0.2 |
| **Sex at birth** |  |  |  | <0.001 |
| Male | 47 (47.0) | 21 (33.0) | 26 (72.0) |  |
| Female | 52 (53.0) | 42 (67.0) | 10 (28.0) |  |
| **Type of residence** |  |  |  | 0.6 |
| Non-permanent | 16 (16.0) | 11 (17.0) | 5 (14.0) |  |
| Permanent | 83 (84.0) | 52 (83.0) | 31 (86.0) |  |
| **Alcohol addiction risk** |  |  |  | 0.8 |
| Low risk | 87 (88.0) | 56 (89.0) | 31 (86.0) |  |
| Medium-High risk | 12 (12.0) | 7 (11.0) | 5 (14.0) |  |
| **Smoking during the last year** |  |  |  | >0.9 |
| Yes | 17 (17.0) | 11 (17.0) | 6 (17) |  |
| No | 82 (83.0) | 52 (83.0) | 30 (83) |  |
| **Body Mass Index (BMI)*** | 26.0 (22.3 - 29.6) | 27.4 (24.3 - 31.2) | 23.2 (20.1 - 26.0) | <0.001 |
| **Comorbidities** |  |  |  |  |
| **Autoimmune diseases** |  |  |  | 0.3 |
| Yes | 4 (4.0) | 4 (6.3) | 0 (0.0) |  |
| **Cancer** |  |  |  | 0.5 |
| Yes | 2 (2.0) | 2 (3.2) | 0 (0.0) |  |
| **Diabetes** |  |  |  | 0.2 |
| Yes | 11 (11.1) | 5 (7.9) | 6 (16.7) |  |
| **Hypertension** |  |  |  | 0.7 |
| Yes | 10 (10.1) | 7 (11.1) | 3 (8.3) |  |
| **Heart Attack** |  |  |  | 0.4 |
| Yes | 1 (1.0) | 0 (0.0) | 1 (2.8) |  |
| **Lung problems** |  |  |  | >0.9 |
| Yes | 12 (12.1) | 8 (12.7) | 4 (11.1) |  |
| **Comorbidities** |  |  |  | >0.9 |
| No | 45 (71.4) | 45 (71.4) | 26 (81.3) |  |
| Yes, at least 1 | 11 (17.5) | 11 (17.5) | 2 (6.3) |  |
| Yes, 2 or more | 7 (11.1) | 7 (11.1) | 4 (12.5) |  |
| **TB history** |  |  |  |  |
| **Current/ Previous TB diagnosis** | |  |  | <0.001 |
| No | 56 (57.0) | 56 (89.0) | 0 (0.0) |  |
| Yes | 43 (43.0) | 7 (11.0) | 36 (100.0) |  |
| **Current /Previous Pulmonary TB treatment** | |  |  | <0.001 |
| Still under treatment | 34 (79.0) | 0 (0.0) | 34 (79.0) |  |
| Did not start treatment | 1 (2.3) | 0 (0.0) | 1 (2.8) |  |
| Did not complete treatment | 1 (2.3) | 1 (14.0) | 0 (0.0) |  |
| Yes, 6 months | 5 (12.0) | 4 (57.0) | 1 (2.8) |  |
| Yes, 9 months | 2 (4.7) | 2 (29.0) | 0 (0.0) |  |
| **COVID-19 history** |  |  |  |  |
| **Vaccine doses** | 3 (3 - 4) | 3 (3 - 4) | 3 (2 - 3) | 0.1 |
| **COVID history** |  |  |  | 0.007 |
| Confirmed | 56 (57.0) | 42 (67.0) | 14 (39.0) |  |
| Very Likely | 43 (43.0) | 21 (33.0) | 22 (61.0) |  |
| **Long COVID** |  |  |  | 0.1 |
| No | 58 (59.0) | 33 (52.0) | 25 (69.0) |  |
| Yes | 41 (41.0) | 30 (48.0) | 11 (31.0) |  |
| *median (IQR); † Fisher’s exact test for categorical variables and Kruskal-Wallis test for numerical variables.  **HHC (Household contacts), ATB (Active TB participants). | | | |  |

**Table S3.** Acute COVID-19 symptoms were reported by the participants in both Peru and Kenya cohorts.

|  |  | **Peru Cohort** | | | | **Kenya Cohort** | | |
| --- | --- | --- | --- | --- | --- | --- | --- | --- |
| **Symptoms** | | **Total, n=99** | **QFT(-)*, n=31** | **QFT(+)*, n=32** | **Active TB*, n=36** | **Total, n=202** | **QFT(-)*, n = 94** | **QFT(+)*, n = 108** |
|  |  | n (%) | n (%) | n (%) | n (%) | n (%) | n (%) | n (%) |
| Cardiopulmonary | Cough | 62 (62.6) | 20 (64.5) | 25 (78.1) | 17 (47.2) | 95 (44.0) | 40 (42.6) | 46 (42.6) |
|  | Shortness of breath | 41 (41.4) | 14 (45.2) | 13 (40.6) | 14 (38.9) | 53 (24.5) | 20 (21.3) | 29 (26.9) |
|  | Chestpain | 38 (38.4) | 14 (45.2) | 14 (43.8) | 10 (27.8) | 59 (27.3) | 22 (23.4) | 32 (29.6) |
|  | Feeling your heart pound or race | 35 (35.4) | 12 (38.7) | 13 (40.6) | 10 (27.8) | 30 (13.9) | 14 (14.9) | 14 (13.0) |
| Constitutional | Feeling feverish | 69 (69.7) | 20 (64.5) | 23 (71.9) | 26 (72.2) | 119 (55.1) | 53 (56.4) | 58 (53.7) |
|  | Chills | 56 (56.6) | 16 (51.6) | 19 (59.4) | 21 (58.3) | 109 (50.5) | 49 (52.1) | 53 (49.1) |
|  | Feeling tired or have low energy | 73 (73.7) | 24 (77.4) | 21 (65.6) | 28 (77.8) | 130 (60.2) | 63 (67.0) | 58 (53.7) |
| Dermatologic | Newspot or rush on the skin | 7 (7.1) | 2 (6.5) | 3 (9.4) | 2 (5.6) | 5 (2.3) | 2 (2.1) | 3 (2.8) |
| Gastrointestinal | Stomach pain | 22 (22.2) | 6 (19.4) | 5 (15.6) | 11 (30.6) | 4 (1.9) | 2 (2.1) | 2 (1.9) |
|  | Nausea gas or indigestion | 22 (22.2) | 6 (19.4) | 10 (31.3) | 6 (16.7) | 19 (8.8) | 12 (12.8) | 7 (6.5) |
|  | Constipation | 10 (10.1) | 4 (12.9) | 3 (9.4) | 3 (8.3) | 3 (1.4) | 1 (1.1) | 2 (1.9) |
|  | Vomiting | 12 (12.1) | 2 (6.5) | 6 (18.8) | 4 (11.1) | 3 (1.4) | 1 (1.1) | 2 (1.9) |
|  | Diarrhea or loose bowel | 28 (28.3) | 13 (41.9) | 7 (21.9) | 8 (22.2) | 8 (3.7) | 7 (7.4) | 1 (0.9) |
| Genitourinary | Trouble with menstrual period | 6 (6.1) | 2 (6.5) | 4 (12.5) | 0 (0.0) | 4 (1.9) | 2 (2.1) | 2 (1.9) |
| Musculoskeletal | Pain in your arms or legs | 39 (39.4) | 14 (45.2) | 13 (40.6) | 12 (33.3) | 65 (35.1) | 28 (29.8) | 33 (30.6) |
|  | Backpain | 56 (56.6) | 18 (58.1) | 19 (59.4) | 19 (52.8) | 23 (10.6) | 11 (11.7) | 11 (10.2) |
| Neurologic | Trouble with smell | 56 (56.6) | 17 (54.8) | 19 (59.4) | 20 (55.6) | 71 (32.9) | 37 (39.4) | 33 (30.6) |
|  | Trouble with taste | 64 (64.6) | 20 (64.5) | 20 (62.5) | 24 (66.7) | 80 (37.0) | 38 (40.4) | 36 (33.3) |
|  | Trouble concentrating, trouble with memory, thinking | 26 (26.3) | 7 (22.6) | 12 (37.5) | 7 (19.4) | 7 (3.2) | 4 (4.3) | 3 (2.8) |
|  | Headache | 75 (75.8) | 20 (64.5) | 26 (81.3) | 29 (80.6) | 93 (43.1) | 41 (43.6) | 44 (40.7) |
|  | Trouble with vision | 27 (27.3) | 8 (25.8) | 8 (25.0) | 11 (30.6) | 7 (3.2) | 3 (3.2) | 4 (3.7) |
|  | Loss of appetite | 52 (52.5) | 16 (51.6) | 19 (59.4) | 17 (47.2) | 62 (28.7) | 29 (30.9) | 31 (28.7) |
|  | Dizziness | 30 (30.3) | 9 (29.0) | 13 (40.6) | 8 (22.2) | 37 (17.1) | 19 (20.2) | 17 (15.7) |
|  | Fainting spells | 1 (1.0) | 0 (0.0) | 0 (0.0) | 1 (2.8) | 2 (0.9) | 0 (0.0) | 2 (1.9) |
|  | Trouble with balance or feeling unsteady | 28 (28.3) | 10 (32.3) | 11 (34.4) | 7 (19.4) | 9 (4.2) | 4 (4.3) | 5 (4.6) |
|  | Numbness, tingling, or “pins and needles” in your arms or legs". | 30 (30.3) | 12 (38.7) | 10 (31.3) | 8 (22.2) | 4 (1.9) | 2 (2.1) | 2 (1.9) |
|  | Trouble sleeping | 42 (42.4) | 13 (41.9) | 18 (56.3) | 11 (30.6) | 33 (15.3) | 14 (14.9) | 18 (16.7) |
| Upper respiratory | Runny nose or congestion | 50 (50.5) | 17 (54.8) | 17 (53.1) | 16 (44.4) | 78 (36.1) | 35 (37.2) | 37 (34.5) |
|  | Sore throat | 60 (60.6) | 20 (64.5) | 22 (68.8) | 18 (50.0) | 90 (41.7) | 37 (39.4) | 46 (42.6) |
| **Active TB: includes persons with TB at different points in antibiotic treatment; QFT (-): TB household contact with negative Quantiferon test; QFT (+): TB household contact with positive QuantiFERON TB Gold test | | | | | | | |  |
|  |  |  |  |  |  |  |  |  |

**A**

**C**


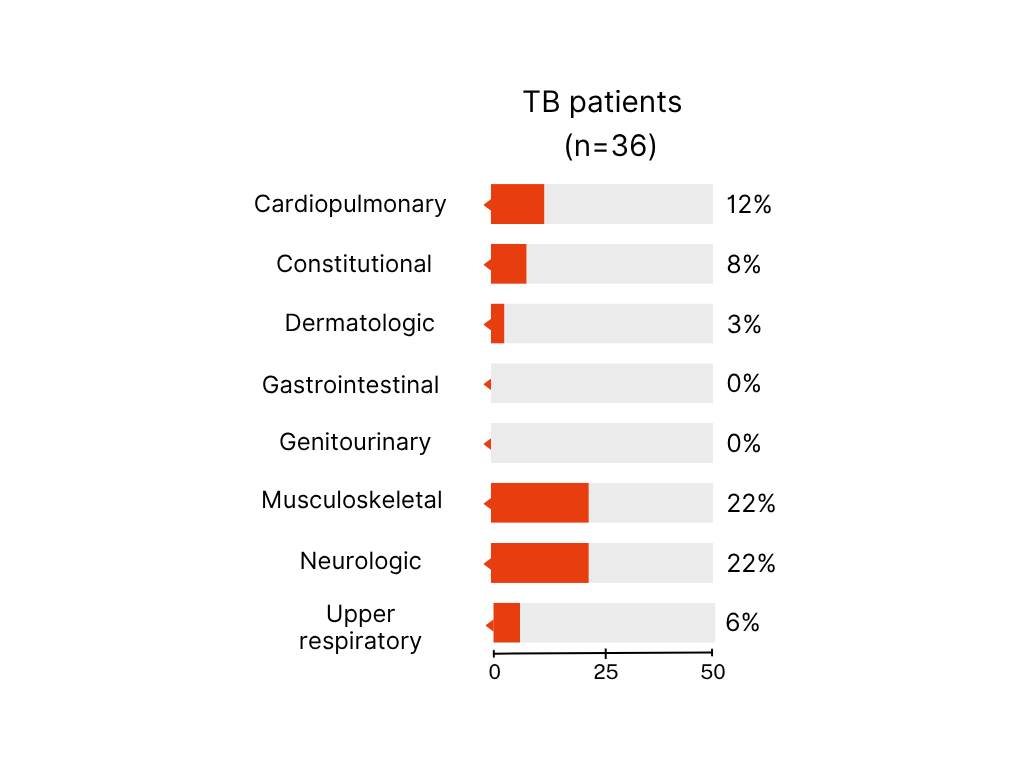

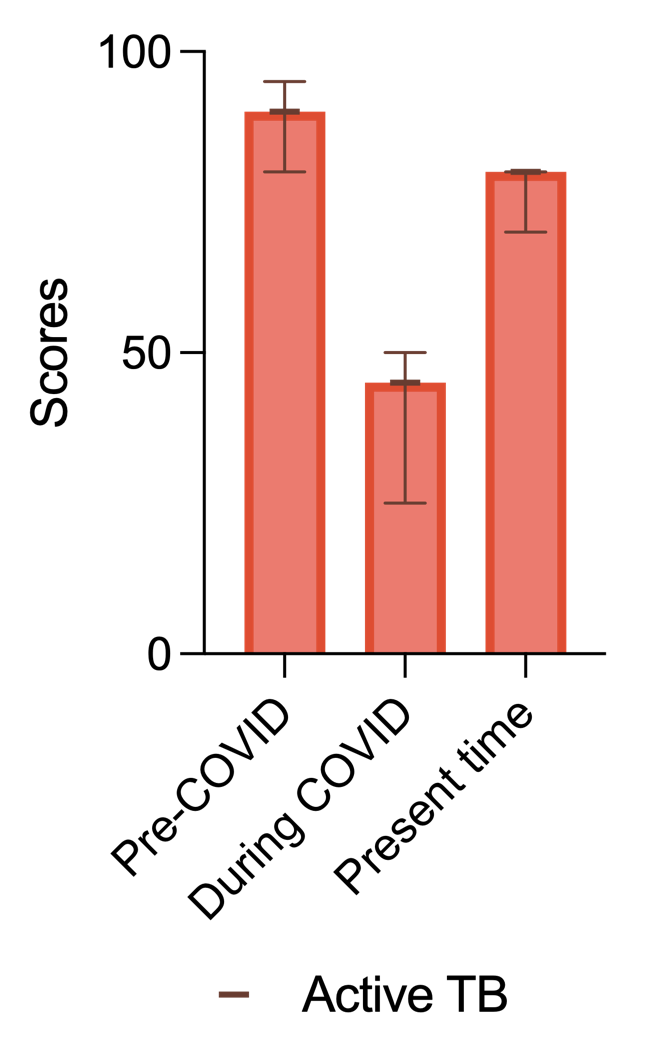


**B**


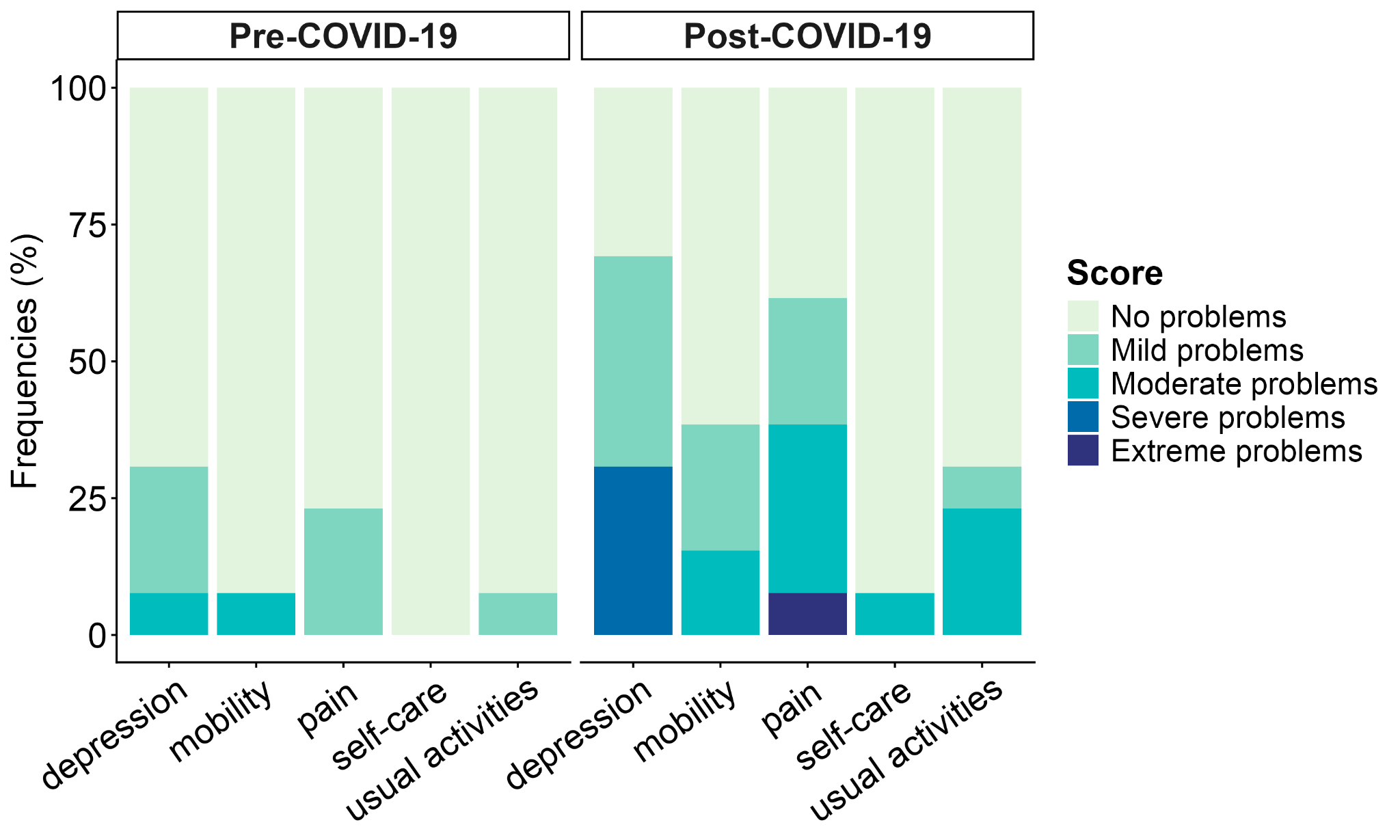


**Figure S1:** LC symptoms and Quality-life responses in the active TB group. A. Frequency of grouped symptoms. B. Comparison between discomfort while performing an activity before and after COVID-19. C. Self-reported life scores.

**Table S4:** Demographic and clinical data and COVID-19 history of Kenyan cohort.

|  | **Kenya Cohort** | | |  |
| --- | --- | --- | --- | --- |
| **Characteristic** | **QFT (-) ****  **n =94 (47%)** | **QFT (+) ****  **n=108 (53%)** |  |  |
|  |  |  | p value† |  |
| **Age*** | 33 (28-42) | 31.5 (25-40) | 0.0725 |  |
| **Sex of birth** |  |  | 0.491 |  |
| Male | 33 (35.1) | 43 (39.8) |  |  |
| Female | 61 (64.9) | 65 (60.2) |  |  |
| **Type of residence** |  |  | 0.767 |  |
| Non-permanent | 2 (2.1) | 3 (2.7) |  |  |
| Permanent | 92 (97.9) | 105 (97.3) |  |  |
| **Alcohol use** |  |  | 0.067 |  |
| Yes | 9 (9.5) | 18 (16.7) |  |  |
| No | 85 (90.5) | 90 (83.3) |  |  |
| **Smoking during last year** |  |  | 0.283 |  |
| Yes | 1 (1.1) | 0 (0.0) |  |  |
| No | 93 (98.9) | 108 (100.0) |  |  |
| **Body Mass Index (BMI)*** | 25.5 (23.1 - 28.1) | 25.2 (22.3 - 28.6) | 0.9299 |  |
| **Comorbidities** | | | | |
| **Autoimmune diseases** |  |  | 0.9 |  |
| No | 93 (98.9) | 107 (99.1) |  |  |
| **Cancer** |  |  | **NC** |  |
| No | 93 (100.0) | 107 (99.1) |  |  |
| **Diabetes** |  |  | **NC** |  |
| No | 93 (100.0) | 107 (99.1) |  |  |
| **Hypertension** |  |  | 0.240 |  |
| No | 89 (94.7) | 107 (99.1) |  |  |
| **Heart Attack** |  |  | **NC** |  |
| No | 93 (100.0) | 107 (99.1) |  |  |
| **Lung problems** |  |  | 0.352 |  |
| No | 93 (98.9) | 107 (99.1) |  |  |
| **Comorbidities** |  |  | 0.3 |  |
| No | 90 (97.0) | 106 (98.0) |  |  |
| Yes, at least 1 | 3 (3.2) | 1 (0.9) |  |  |
| Yes, 2 or more | 0 (0.0) | 1 (0.9) |  |  |
| **COVID-19 history** | | | | |
| **Vaccine doses** | 1 (1 - 1) | 1 (1 - 1) | 0.091 |  |
| **COVID history** |  |  |  |  |
| Confirmed | 94 (100.0) | 108 (100.0) |  |  |
| Very Likely | 0 (0.0) | 0 (0.0) |  |  |
| **Long COVID** |  |  |  |  |
| No | 94 (100.0) | 108 (100.0) |  |  |
| Yes | 0 (0.0) | 0 (0.0) |  |  |
| *Median (IQR); † Fisher’s exact test for categorical variables and Kruskal-Wallis test for numerical variables. **QFT (-): TB household contact with negative QuantiFERON test; QFT (+): TB household contact with positive QuantiFERON TB Gold test; NA: Not applicable; NC: Not calculated | | | | |


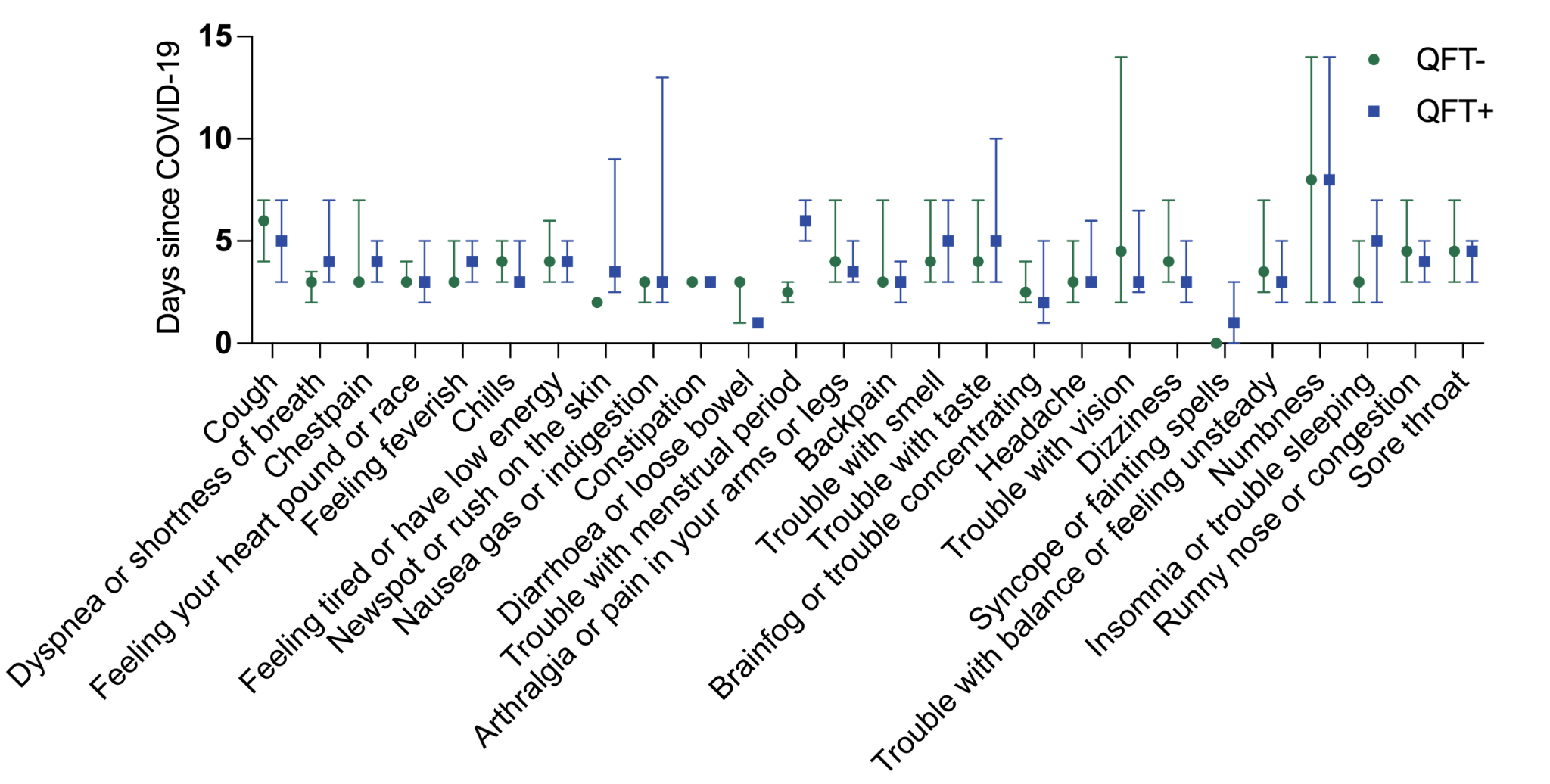


**Figure S2:** The median duration of COVID-19 symptoms by QFT+ and QFT- in Kenya.

**A.**

**
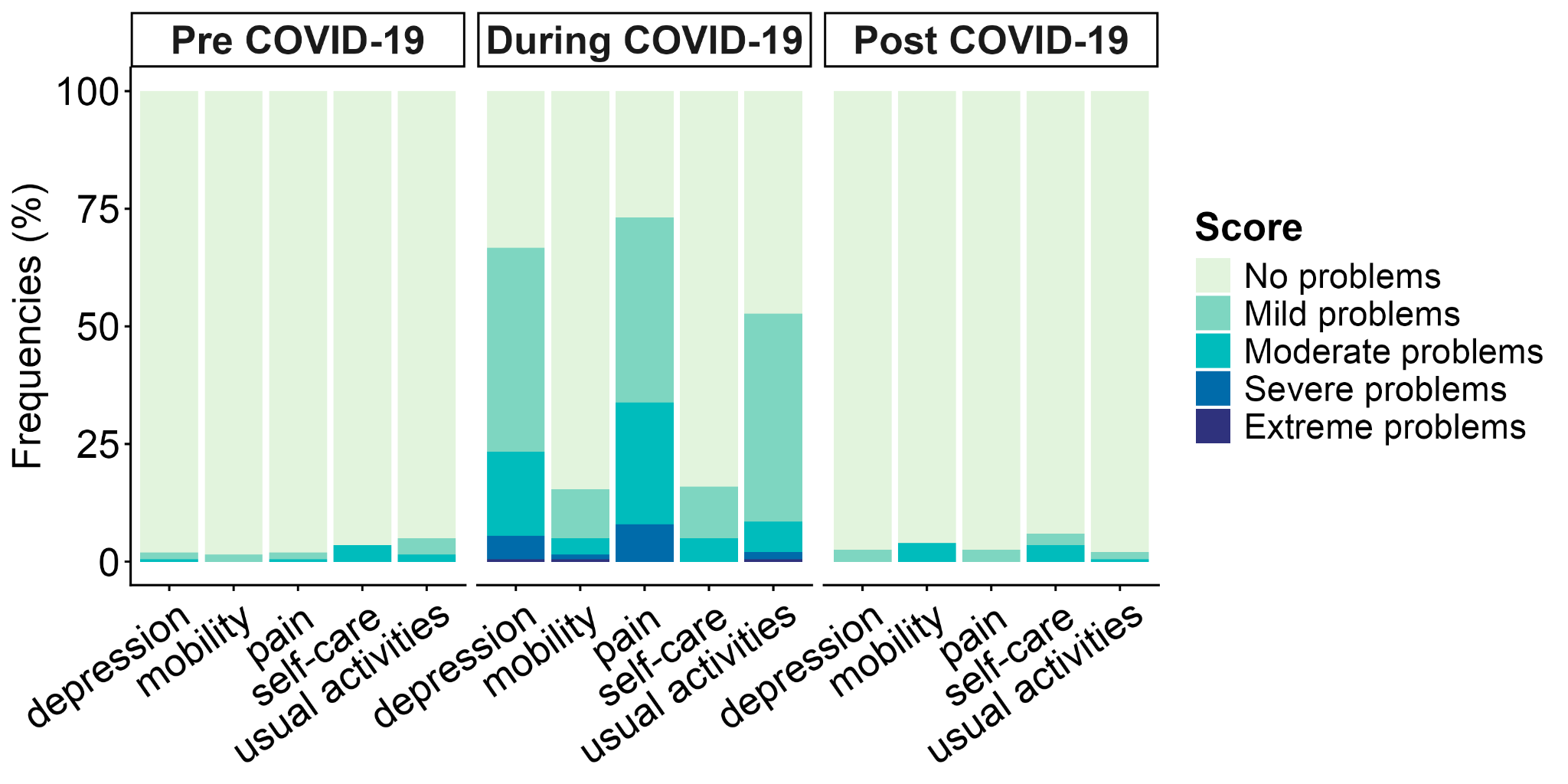
**

**B.**

**
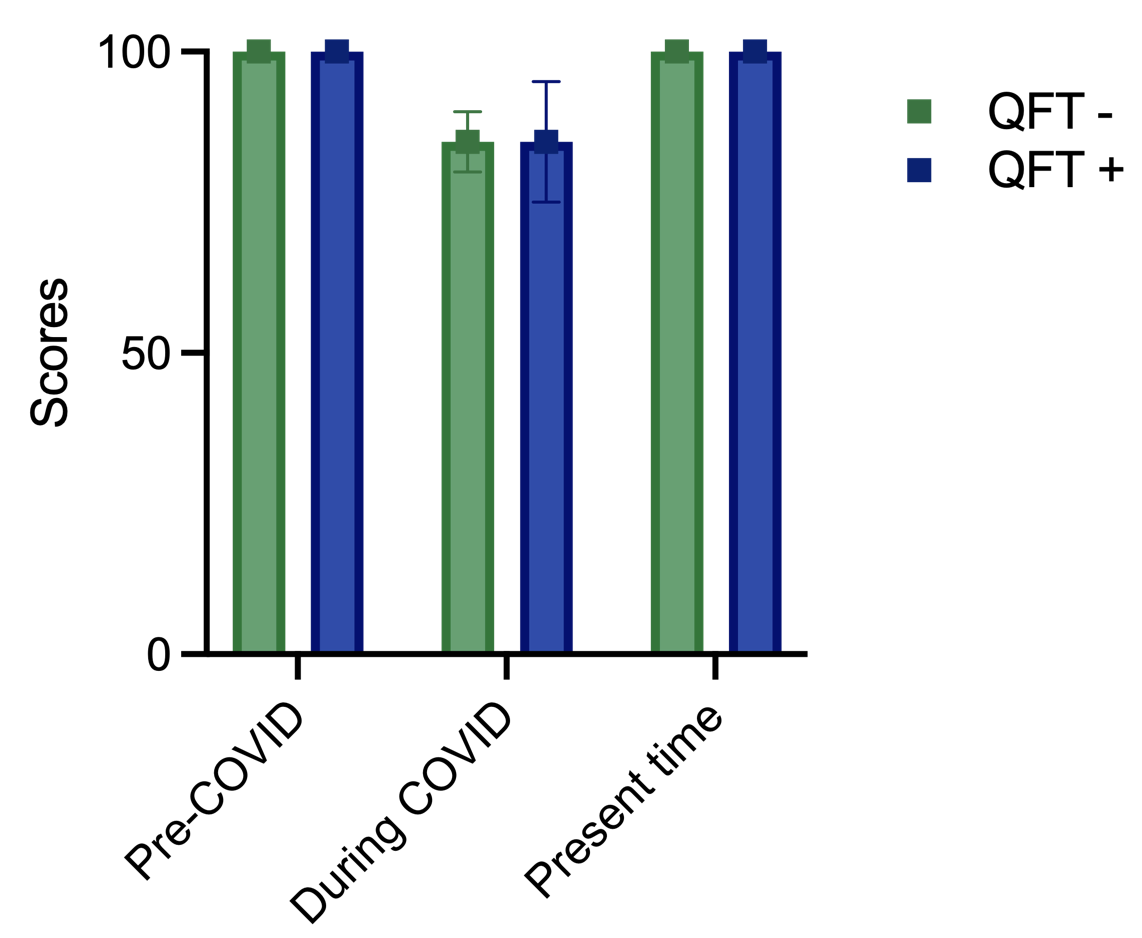
**

**Figure S3:** Quality of Life in Kenyan cohort. A. Pre COVID-19, during COVID-19 and Post COVID-19 prevalence of difficulties found while performing an activity in the Kenyan Cohort. B. Self-reported health scores in the Kenyan Cohort.
